# Impact of workplace discrimination and harassment among National Health Service staff working in London trusts: results from the TIDES study

**DOI:** 10.1192/bjo.2020.137

**Published:** 2020-12-16

**Authors:** Rebecca D. Rhead, Zoe Chui, Ioannis Bakolis, Billy Gazard, Hannah Harwood, Shirlee MacCrimmon, Charlotte Woodhead, Stephani L. Hatch

**Affiliations:** Department of Psychological Medicine, King's College London, UK; Department of Psychological Medicine, King's College London, UK; Centre for Implementation Science, Health Services and Population Research Department and Department of Biostatistics and Health Informatics, Institute of Psychiatry, Psychology and Neuroscience, King's College London, UK; Department of Psychological Medicine, King's College London, UK; Department of Psychological Medicine, King's College London, UK; Department of Psychological Medicine, King's College London, UK; Department of Psychological Medicine, King's College London; and ESRC Centre for Society and Mental Health, King's College London, UK; Department of Psychological Medicine, King's College London; and ESRC Centre for Society and Mental Health, King's College London, UK

**Keywords:** Discrimination, harassment, heathcare, mental health, inequalities

## Abstract

**Background:**

Harassment and discrimination in the National Health Service (NHS) has steadily increased over the past 5 years with London being the worst performing region. There is a lack of data and research on the impact this is having on staff health and job satisfaction. Such data are necessary to inform the development of effective workplace interventions to mitigate the effects these experiences have on staff.

**Aims:**

Examine the impact of harassment and discrimination on NHS staff working in London trusts, utilising data from the 2019 TIDES cross-sectional survey.

**Method:**

In total, 931 London-based healthcare practitioners participated in the TIDES survey. Regression analysis was used to examine associations between the sociodemographic characteristics of participants, exposure to discrimination and harassment, and how such exposures are associated with physical and mental health, job satisfaction and sickness absence.

**Results:**

Women, Black ethnic minority staff, migrants, nurses and healthcare assistants were most at risk of discrimination and/or harassment. Experiencing either of the main exposures was associated with probable anxiety or depression. Experiencing harassment was also associated with moderate-to-severe somatic symptoms. Finally, both witnessing and experiencing the main exposures were associated with low job satisfaction and long periods of sickness absence.

**Conclusions:**

NHS staff, particularly those working in London trusts, are exposed to unprecedented levels of discrimination and harassment from their colleagues. Within the context of an already stretched and under-resourced NHS, in order to combat poor job satisfaction and high turnover rates, the value of all healthcare practitioners must be visibly and continuously reinforced by all management and senior leaders.

## Background

Reported incidents of workplace discrimination and bullying, harassment and abuse (BHA) from National Health Service (NHS) staff have steadily increased over the past 5 years.^1^ Both NHS staff survey and workforce equality reports have consistently found staff from ethnic minority groups to be the most vulnerable and suffer disproportionate amounts of abuse.^1,2^ Workplace discrimination and harassment is illegal and has been shown to have detrimental, sometimes devastating, effects on employees.^3,4^ Although there are ongoing efforts within the NHS to monitor workplace inequality, there is a lack of data and research on the impact of workplace harassment and discrimination from colleagues on their health and job satisfaction. Such data are necessary to inform the development of effective workplace interventions to mitigate the growing problem of discrimination and BHA in the NHS.

## BHA

The term BHA is used in this study to refer to three similar but distinct concepts. Harassment is defined under the Equality Act 2010 as ‘Unwanted conduct related to a relevant protected characteristic, which has the purpose or effect of violating an individual's dignity or creating an intimidating, hostile, degrading, humiliating or offensive environment for that individual’.^5^ Bullying is generally defined as any behaviour that is offensive, intimidating, malicious or insulting, similarly workplace abuse is behaviour that causes workers emotional or physical harm. Harassment and bullying are forms of workplace abuse. Neither bullying nor abuse are formally identified within the Equality Act 2010, but many of the characteristics are the same as harassment.

## Discrimination

Discrimination, a separate and distinct concept from BHA, is defined as treating a person unfairly because of who they are or because they possess any of the nine characteristics protected by law (age, gender, disability, gender reassignment, marriage/civil partnership, pregnancy/maternity, race, religion or belief, sexual orientation).^6^ General workplace BHA against an individual, and acts of discrimination against individuals or groups in the workplace, are associated with greater psychological distress, anxiety and depression^7,8^ as well as poorer physical health.^9,10^ All of which are in turn associated with low job satisfaction^11^ and, in a study of hospital staff in Finland, increased sickness absence.^12^

## Sickness absence

The link between both types of exposures and sickness absence is not necessarily only mediated by poor health. Staff may take or prolong sickness absence to avoid a negative work environment or low job satisfaction as a result of feeling undervalued.^13,14^ It is also important to consider that the aforementioned health or job performance outcomes are not necessarily precipitated by direct personal experiences of discrimination or BHA. There is evidence to suggest that witnessing such incidents also contributes to a negative work environment and is sufficient enough to be detrimental to one's health,^15^ although further study is required to establish the nature and strength of association between witnessing and health.

## Racial and ethnic inequalities in the workplace

Several studies have highlighted the abuse and discrimination experienced by Black, ethnic minority and migrant nurses from their colleagues,^16–19^ as well as being continually undervalued, ignored for promotion and excessively disciplined by their managers.^18^ Such acts of staff-on-staff discrimination and BHA exemplifies that, as well as being experienced from patients, discrimination and BHA can occur within the hierarchy of hospital staff horizontally (between staff in a single hierarchy) and vertically (between staff on a similar level).

The NHS Equality & Diversity Council conceived the Workforce Race Equality Standard (WRES) in 2014 – a programme used to gauge race equality. Although the WRES (now mandated throughout the NHS) monitors workplace inequality by assessing workforce and NHS staff survey data as well as the representativeness and diversity of leadership roles, it is unable to establish the effects that discrimination and BHA have on staff outcomes. The WRES is also unable to determine inequalities by important demographic information such as migration status, as such data is not available. Finally, the extent to which experiencing discrimination and BHA from colleagues has an impact on the health and well-being of NHS staff specifically is unknown.

Although this is a national problem, it is particularly prevalent in London NHS trusts. Despite having the most ethnically diverse workforce, and the greatest proportion of migrants, London is the worst performing English region for staff-on-staff BHA (30.9% among Black and ethnic minority staff and 27.8% among staff from White ethnic groups) and discrimination (16.4% among Black and ethnic minority staff and 8.4% among White ethnic groups). London is also the only region to have observed a sustained increase in both since 2015.^20^ If left unchecked, this will likely lead to poor staff retention for the NHS, as well as diminished quality of life and limited and inequitable career progression for staff.^21^ Additionally, this may have negative consequences for the quality of patient care, jeopardising patients’ safety.^22,23^ Robust investigations are urgently needed to clearly identify those most at risk and how it is having an impact on their health and ability to work.

## Aims

Utilising data from the TIDES (Tackling Inequalities and Discrimination in Healthcare Services) survey, this study examines the impact of discrimination and BHA among NHS staff working in London trusts and the factors associated with negative outcomes. Specifically, this study aims to:

- Identify the sociodemographic characteristics associated with exposure to discrimination and BHA from staff.
- Examine if discrimination and BHA is associated with: poor physical and mental health; and job satisfaction and sickness absence.

## Method

### Data

This study used data from an online survey conducted as part of the TIDES study^24^ an ongoing investigation into how discrimination experienced by both patients and healthcare practitioners (HCPs) may generate and perpetuate inequalities in health service use. As part of the TIDES study, a survey of HCPs working in London trusts was conducted to assess how discrimination and related factors contribute to inequalities in the health service domain.

In the absence of an available NHS staff sampling frame and the need for the study to maintain independence from the NHS, given the sensitive nature of the adversities, HCPs (doctors, nurses, healthcare assistants and Improving Access to Psychological Therapy (IAPT) workers) were recruited directly from higher education institutions and NHS trusts across London. Gatekeepers (used to access HCPs) consisted of foundation managers, training programme directors, department heads, preceptorship module leaders, programme directors and IAPT service managers. With the aid of gatekeepers, participants were recruited via (a) training and education sessions, (b) email circulars, and (c) social media and the TIDES study website.

Researchers verified eligibility, which was restricted to HCPs over the age of 18 years working or training in a London NHS trust with at least 12 months’ experience of working or training in a clinical setting with direct patient contact. Throughout data collection, demographic characteristics of the participants were monitored to ensure that the sample reflected the diversity of the NHS staff population in London in terms of gender, ethnicity and occupational group (using data from the NHS staff survey, 2018^2^). For more information on recruitment please see Supplementary document 1, available at https://doi.org/10.1192/bjo.2020.137.

A total of 931 participants were recruited between February 2018 and August 2019 across 33 of the 34 London trusts.

### Ethical approval

This study received ethical approval from King's College London Research Ethics Committee for Psychiatry, Nursing and Midwifery (REC reference: HR-17/18–4629; IRAS project ID: 230692).

### Patient and public involvement

The study was supported by an advisory group which provided input to the programme of research. This advisory group met on a regular basis for the duration of the study. At the end of the study, the advisory group commented on the findings and contributed to the dissemination plan.

### Sociodemographic characteristics

The sociodemographic characteristics of participants were examined using self-reported data on gender, age category (19–30, 31–40, 41–50, ≥51 years), occupational group (medical/dental, allied health professionals/psychological therapists, nurses, healthcare assistants and other professions (service managers, discharge coordinators, patient outcomes manager and medical secretaries)), pay band (high, pay band 6–8; medium, pay band 5; low, pay band 2–4; and student, no pay band), sexual orientation (heterosexual, non-heterosexual (gay or lesbian, bisexual, other)) ethnic group (based on census category: White British, White other, Black, Asian and mixed) and migration status (migrant, non-migrant).

### Discrimination and BHA

The two main exposures of this study are (a) discrimination and (b) BHA – perpetrated by colleagues, as opposed to patients/families. Both witnessing and experiencing these acts were assessed in this study. Measures of witnessing and experiencing both main exposures were taken from the WRES so that comparisons with the WRES findings could be made.

To assess discrimination, participants were asked ‘In the last 12 months have you personally experienced discrimination at work from a manager/team leader or other colleagues’ (no, 0; yes, 1) and whether ‘In the last 12 months have you witnessed discrimination against colleagues from any of the following? Manager/team leader or other colleagues’ (no, 0; yes, 1).

To assess BHA, participants were asked ‘In the last 12 months how many times have you personally experienced BHA from managers?’ and ‘In the last 12 months how many times have you personally experienced BHA from other colleagues?’ – these data were combined and dichotomised to produce a single measure of whether the participant had experienced harassment from any co-worker (neither, 0; either, 1). Participants were also asked ‘In the last 12 months have you witnessed BHA of a colleague by a manager or other colleagues?’ (no, 0; yes, 1).

### Mental and physical health

In our assessment of the health problems associated with harassment and discrimination, meeting criteria for common mental disorder (probable anxiety or depression) and somatic symptoms were assessed.

The Patient Health Questionnaire (PHQ-9) was used to assess depressive symptoms with a score of ≥10 suggestive of a likely diagnosis of depressive disorder and therefore indicative of caseness.^25^ A PHQ-9 score ≥10 has a sensitivity of 88% and a specificity of 88% for major depression. Both the internal consistency and test–retest reliability of the PHQ-9 is excellent (Cronbach α = 0.89, intraclass correlation (ICC) = 0.84).

The Generalised Anxiety Disorder (GAD-7) was used to assess anxiety symptoms with a score of ≥8 suggestive of a likely diagnosis of anxiety disorder and therefore indicative of caseness.^26^ The internal consistency of the GAD-7 is excellent (Cronbach α = 0.92). Test–retest reliability is also good (ICC = 0.83). A score of ≥8 has a sensitivity of 89% and a specificity of 82%.

When assessing the association between the main exposures on mental health, a combined measure of anxiety and depression was used to indicate the presence of a probable anxiety or depression (scoring above the threshold for either measure, 1; below threshold for both, 0).

Somatisation symptoms were measured using the somatic symptom module of the PHQ-15.^27^ The PHQ-15 is a somatic symptom subscale derived from the full PHQ.^28^ Participants were asked to rate the severity of 15 physical symptoms as 0 (‘not bothered at all’); 1 (‘bothered a little’); or 2 (‘bothered a lot’). Total PHQ-15 scores ranged from 0 to 30, a cut-off score of 10 or more was used to indicate moderate-to-severe somatic symptoms.

Participants were also asked if they had any long-standing health problems, illness or disability (0, none; 1, at least one). This was adjusted for in our examination of probable anxiety or depression and somatic symptoms.

### Job satisfaction and sickness absence

Reported sickness absence was assessed using an item from the World Health Organization's Health and Work Performance Questionnaire – a self-report instrument designed to estimate the workplace costs of health problems in terms of reduced job performance, sickness absence and work-related accidents/injuries.^29^ The item used in this study asked participants to indicate whether they have taken a 2-week leave of sickness absence in the past year (0, no; 1, yes).

Our measure of job satisfaction was an item taken from the Non-Illness Predictors of Sickness Absence Questionnaire^30^ – a scale designed to measure non-illness predictors of sickness absence. Participants were asked to indicate on a 5-point Likert scale (1, strongly disagree, to 5, strongly agree) if all things considered they were satisfied with their job. This data was dichotomised to produce a binary measure of job satisfaction (not satisfied, 1–3; satisfied, 4–5).

### Analysis

All analysis was conducted in Stata 15.^31^ Survey weights were calculated using iterative proportional fitting (IPF) using the ipfraking package (18), to align TIDES survey data more closely to that of the population (HCPs working in London trusts). IPF was used over the more commonly used post-stratification approach as only marginal population distributions were known (IPF is a way to approximate post-stratification on a set of variables when only their marginal population distributions are known).^32^

To do this, control variables (marginal population data) on gender and ethnicity were obtained from NHS workforce records via NHS Digital. IPF is an iterative process and control variables were assessed in sequence to produce a calibrated weight variable. Sexual orientation, occupational group and migrant status were not weighted for because of insufficient control data. Although control data on age was available, it was not possible to weight by age group as the difference between sample and population data was too great (TIDES survey was considerably more skewed towards younger HCPs). See Supplementary Table 1 for further detail. Following the calculation of survey weights, the prevalence of witnessing or experiencing the two main exposures (discrimination and BHA) were calculated.

Logistic regression models were used to (a) determine whether discrimination, and BHA are associated with participant characteristics (gender, age, ethnicity, migrant status, sexual orientation and occupational group); (b) assess the association between discrimination, and BHA and health (probable anxiety or depression and somatic symptoms); and (c) assess how the main exposures are associated with job satisfaction and recent sickness absence.

All analyses were adjusted for sociodemographic characteristics and long-standing health problems, illness or disability. All proportions, odds ratios (ORs) and 95% CIs reported in this study are weighted (unless stated otherwise), frequencies are unweighted.

## Results

A total of 931 participants completed the TIDES survey. Sexual orientation was not reported by 26 participants (2.8%). As a result, the sample size was reduced to 905 for models that accounted for sexual orientation.

### Sociodemographic characteristics and discrimination and harassment exposures

As shown in Table 1, overall, 21% of the participants experienced discrimination and 44% experienced BHA from colleagues. Women were more likely to experience discrimination (24% *v.* 11%, OR = 2.45, 95% CI 1.45–4.12) and harassment (46% *v.* 36%, OR = 1.52, 95% CI 1.07–2.16) than men, and HCPs from Black ethnic groups were more likely to experience and witness both exposures compared with the White British group. Asian HCPs were also proportionately more likely to experience discrimination compared with White British respondents. Migrants were proportionately more likely to witness, and experience discrimination and experience harassment compared with non-migrants. Finally, nurses, healthcare assistants and other professions were more likely to experience discrimination than medical staff.

**Table 1:** Sociodemographic characteristics and probable mental health problems associated with discrimination and BHA among healthcare practitioners^a^

	Discrimination								BHA									
	Experiencing				Witnessing				Experiencing				Witnessing				Total	
	*n*	%	OR	95% CI	*n*	%	OR	95% CI	*n*	%	OR	95% CI	*n*	%	OR	95% CI	*n*	%
Overall	195	21	–	–	161	17	–	–	413	44	–	–	239	26	–	–	–	–
Gender																		
Male	18	11	1.00	1.00–1.00	26	15	1.00	1.00–1.00	59	36	1.00	1.00–1.00	41	25	1.00	1.00–1.00	167	24
Female	177	24	2.45	1.45–4.12	135	18	1.22	0.77–1.95	354	46	1.52	1.07–2.16	198	26	1.06	0.72–1.58	764	76
Age group (years)																		
<25	35	13	1.00	1.00–1.00	26	10	1.00	1.00–1.00	94	37	1.00	1.00–1.00	47	19	1.00	1.00–1.00	251	27
25–34	78	17	1.33	0.86–2.07	77	17	1.83	1.13–2.97	198	43	1.27	0.92–1.75	116	26	1.50	1.02–2.22	449	48
35–44	47	32	3.06	1.84–5.10	35	25	3.02	1.70–5.34	73	52	1.83	1.20–2.81	44	31	1.99	1.22–3.24	142	16
45	35	39	4.13	2.34–7.30	23	27	3.32	1.75–6.30	48	53	1.89	1.14–3.13	32	38	2.66	1.53–4.62	89	10
Ethnicity																		
White British	59	14	1.00	1.00–1.00	57	13	1.00	1.00–1.00	174	41	1.00	1.00–1.00	100	24	1.00	1.00–1.00	421	44
White other	20	18	1.31	0.74–2.32	21	20	1.60	0.91–2.80	55	49	1.43	0.93–2.21	24	22	0.91	0.54–1.51	107	11
Black	62	35	3.08	2.00–4.74	42	24	2.04	1.30–3.21	91	52	1.58	1.10–2.25	56	33	1.56	1.05–2.31	179	22
Asian	42	24	1.95	1.23–3.08	30	17	1.35	0.82–2.20	70	41	1.03	0.71–1.49	43	25	1.05	0.69–1.60	166	21
Mixed	12	20	1.63	0.81–3.28	11	21	1.69	0.82–3.49	23	39	0.93	0.53–1.65	15	26	1.16	0.61–2.20	57	4
Migrant status																		
Non-migrant	99	16	1.00	1.00–1.00	89	14	1.00	1.00–1.00	251	41	1.00	1.00–1.00	144	24	1.00	1.00–1.00	603	63
Migrant	96	29	2.08	1.50–2.89	72	22	1.71	1.20–2.43	162	49	1.36	1.03–1.80	95	30	1.35	0.99–1.85	328	37
Sexual orientation																		
Heterosexual	172	21	1.00	1.00–1.00	134	17	1.00	1.00–1.00	347	43	1.00	1.00–1.00	201	25	1.00	1.00–1.00	806	88
Non-heterosexual	17	16	0.69	0.39–1.21	22	20	1.26	0.75–2.12	50	49	1.30	0.84–2.00	29	28	1.11	0.69–1.79	99	12
Occupational group																		
Medical	16	10	1.00	1.00–1.00	25	16	1.00	1.00–1.00	57	39	1.00	1.00–1.00	39	26	1.00	1.00–1.00	138	17
Allied health professional/ psychological therapy	16	17	1.88	0.87–4.05	20	21	1.43	0.73–2.81	29	30	0.69	0.39–1.23	26	29	1.19	0.65–2.18	93	10
Nurse	128	22	2.60	1.47–4.61	95	16	1.03	0.63–1.70	279	47	1.41	0.96–2.07	146	25	0.96	0.63–1.48	600	65
Healthcare assistant	23	31	4.22	2.02–8.81	13	19	1.22	0.57–2.60	34	45	1.31	0.73–2.35	20	27	1.07	0.56–2.05	75	8
Other	12	48	8.49	3.24–22.28	8	33	2.58	0.98–6.78	14	53	1.79	0.74–4.30	8	33	1.41	0.55–3.60	25	0

BHA, bullying, harassment and abuse.

a. Numbers (*n*), weighted percentages (%), odds ratios (OR) and 95% CIs are shown.

### Associations of exposures with probable anxiety or depression and somatic symptoms

Table 2 shows the associations between the two main exposures (discrimination, and BHA – both witnessing or experiencing) with probable anxiety or depression and somatic symptoms. The overall sample prevalence of probable anxiety or depression was 22%.

**Table 2:** Regression analysis to show probable anxiety or depression and somatic symptoms associated with experiencing discrimination and BHA^a^

			Model 1^b^		Model 2^c^		Model 3^d^	
	*n*	%	OR	95% CI	OR	95% CI	OR	95% CI
*Probable anxiety or depression*								
Discrimination								
Experienced								
No	149	20	1.00	–	1.00	–	1.00	–
Yes	54	27	1.50	1.04–2.17	1.87	1.23–2.85	1.74	1.14–2.67
Witnessed								
No	163	21	1.00	–	1.00	–	1.00	–
Yes	40	24	1.21	0.81–1.82	1.50	0.97–2.31	1.31	0.83–2.06
BHA								
Experienced								
No	88	16	1.00	–	1.00	–	1.00	–
Yes	115	28	1.93	1.40–2.67	2.13	1.51–3.00	1.96	1.38–2.79
Witnessed								
No	146	21	1.00	–	1.00	–	1.00	–
Yes	57	23	1.17	0.82–1.67	1.36	0.92–2.01	1.26	0.85–1.86
*Somatic symptoms (moderate–severe)*								
Discrimination								
Experienced								
No	230	30	1.00	–	1.00	–	1.00	–
Yes	83	42	1.68	1.21–2.34	1.71	1.16–2.51	1.38	0.92–2.08
Witnessed								
No	243	30	1.00	–	1.00	–	1.00	–
Yes	70	42	1.68	1.18–2.39	2.04	1.36–3.04	1.76	1.14–2.70
BHA								
Experienced								
No	139	25	1.00	–	1.00	–	1.00	–
Yes	174	41	2.00	1.51–2.66	2.17	1.59–2.96	1.78	1.28–2.48
Witnessed								
No	221	31	1.00	–	1.00	–	1.00	–
Yes	92	37	1.33	0.97–1.81	1.44	1.02–2.04	1.31	0.90–1.89

BHA, bullying, harassment and abuse.

a. Numbers (*n*), weighted percentages (%), odds ratios (OR) and 95% CIs are shown.

b. Model 1: crude.

c. Model 2: adjusted for age, gender, ethnicity, migrant status, sexual orientation, occupational role and pay band.

d. Model 3: adjusted for age, gender, ethnicity, migrant status, sexual orientation, occupational role, pay band and long-standing health problems illness or disability (the somatic symptom model is also adjusted for probable anxiety or depression).

We found that experiencing (but not witnessing) both discrimination (OR = 1.50, 95% CI 1.04–2.17) and BHA (OR = 1.93, 95% CI 1.40–2.67) were significantly associated with probable anxiety or depression and that associations persisted after adjusting for sociodemographic characteristics and long-standing health problems, illness or disability.

Experiencing BHA (OR = 2.00, 95% CI 1.51–2.66) and witnessing discrimination (OR = 1.68, 95% CI 1.18–2.39) were found to be significantly associated with moderate or severe somatic symptoms. These associations persisted after adjusting for sociodemographic characteristics, long-standing health problems, illness or disability and probable anxiety or depression. Although experiencing discrimination was also associated with somatic symptoms, the association became non-significant following adjustments (model 3, Table 2).

### Associations of exposures with job satisfaction and sickness absence

As shown in Table 3, participants who reported experiencing discrimination (OR = 2.32, 95% CI 1.49–3.62) or BHA (OR = 2.21, 95% CI 1.46–3.36) in the workplace had approximately double the odds of having taken a minimum 2-week sickness absence in the past year compared with those who did not after adjusting for sociodemographic characteristics, and long-standing health problems, illness or disability (model 3, Table 3).

**Table 3:** Regression analysis to show how sickness absence and job satisfaction are associated with discrimination and BHA^a^

	*n*	%	Model 1		Model 2		Model 3	
			OR	95% CI	OR	95% CI	OR	95% CI
*Sickness absence (2 weeks in past year)*								
Discrimination – experienced								
No	72	9	1.00	1.00–1.00	1.00	1.00–1.00	1.00	1.00–1.00
Yes	37	19	2.32	1.49–3.62	2.29	1.41–3.74	1.98	1.18–3.34
BHA – experienced								
No	347	58	1.00	1.00–1.00	1.00	1.00–1.00	1.00	1.00–1.00
Yes	66	39	2.21	1.46–3.36	2.48	1.60–3.85	2.11	1.34–3.30
*Job satisfaction*								
Discrimination – experienced								
No	555	74	1.00	1.00–1.00	1.00	1.00–1.00	1.00	1.00–1.00
Yes	101	52	0.37	0.26–0.51	0.38	0.26–0.54	0.38	0.27–0.55
BHA – experienced								
No	162	60	1.00	1.00–1.00	1.00	1.00–1.00	1.00	1.00–1.00
Yes	237	37	0.40	0.29–0.53	0.39	0.29–0.54	0.39	0.29–0.54

BHA, bullying, harassment and abuse.

a. Numbers (*n*), weighted percentages (%), odds ratios (OR) and 95% CIs are shown.

Model 1: crude.

Model 2: adjusted for age, gender, ethnicity, migrant status, sexual orientation, occupational role and pay band.

Model 3: adjusted for age, gender, ethnicity, migrant status, sexual orientation, occupational role, pay band and long-standing health problems, illness or disability.

Job satisfaction was significantly lower for participants who had experienced discrimination (OR = 0.37, 95% CI 0.26–0.51) or BHA (OR = 0.40, 95% CI 0.29–0.53). The strength and direction of the associations remained largely unchanged after adjusting for sociodemographic characteristics and long-standing health problems, illness or disability (model 3, Table 3).

## Discussion

Focusing on NHS HCPs working in London – the most prevalent region for discrimination and BHA in England – this is the first UK study to use survey data to identify healthcare staff most likely to experience or witness such incidents, and to establish associated health and work outcomes. Such an investigation is urgently needed given the increasing prevalence of such incidents as well as the lack of existing data to assess the impact this has on the NHS workforce.

Such research is particularly urgent in light of the coronavirus disease 2019 (COVID-19) pandemic, which has likely exacerbated working conditions for NHS staff from Black and ethnic minority backgrounds who, as well as having to navigate greater exposure to discrimination and BHA, are particularly vulnerable to COVID-19,^33,34^ work in roles with increased exposure to the disease^35^ and experience disempowerment in relation to complaining about deleterious working conditions.^20^ These racial and ethnic disparities need to be addressed if we are to avoid the social, economic and moral costs of excessive adverse mental health and occupational outcomes for Black, Asian and ethnic minority staff.

### Sociodemographic characteristics associated with BHA

We found that the overall prevalence of workplace discrimination and harassment (from colleagues or other staff) was higher in the TIDES survey (discrimination 20%, harassment 41%) compared with recent NHS reports for staff working in London trusts (discrimination 12%, harassment 19%).^2^ This disparity is likely attributable to TIDES being independent of the NHS, with participants less likely to report such exposures in a survey being administrated on behalf of their employer. It is also possible that those who have experienced discrimination or harassment may have been more likely to take part in the TIDES survey than the NHS staff survey.

Our results indicate that both nurses and healthcare assistants were more likely to experience discrimination than medical staff (the designated reference category because of their senior position in the hierarchy of NHS staff). This is possibly reflective of how nurses and healthcare assistants are exposed to vertical as well as horizontal discrimination, i.e. they may experience discrimination from colleagues in higher positions of the workplace hierarchy, such as medical staff (vertical), in addition to potentially experiencing discrimination from within their own staff group (horizontal).^36^

Vertical discrimination and BHA evoke differences in relational power across occupational hierarchy,^37^ whereas horizontal discrimination and BHA may affect cohesion and cooperation between colleagues. For the latter, staff exclude or belittle others, limiting career development opportunities such as access to support systems and networking events. This often leads to staff leaving their job allowing these systemic issues to continue.

Horizontal discrimination and BHA for nurses may be born out of the extra pressure this occupational group faces – they have low control and autonomy (because of lower banding) – as well as with high responsibility for patient safety. Experiencing discrimination and BHA from peers may also exacerbate the impact of other systemic/vertical inequalities.^38^ Both are likely to affect segregation across the workplace, vertically in terms of distribution of senior roles and horizontally across different workplaces.

Ethnicity and migrant status also factor into these findings, for example, healthcare assistants had the greatest proportion of migrants (56%) and non-White ethnic groups (62%) compared with any other HCP role. Nurses who reported discrimination mostly belonged to the Black ethnic group (38%). Women, Black ethnic minority staff and migrants were also identified as the members of staff most at risk of discrimination and/or BHA.^36,39^ This highlights the importance of considering both migration status and ethnicity – examining the latter without considering the former when examining workforce inequalities is likely to obscure important contextual findings and diminish the experiences of migrants. Our findings here also echo previous qualitative studies that have found that nurses from Black ethnic groups as well as migrant nurses often experience BHA from both their colleagues and superiors.^36,40^ Additional research is required to assess the intersections between ethnicity and migration status by quantifying the magnitude of the effect migrant status has as a moderator on associations between ethnicity and negative outcomes.

Black ethnic minority staff are greatly underrepresented in the most senior jobs in NHS trusts^41^ and have been found here to be one of the groups at most risk of experiencing both BHA and discrimination. This form of vertical discrimination must be addressed – by increasing the proportion of Black, Asian and ethnic minority groups in leadership roles – in order to combat the systemic inequalities across the NHS.^42^

### Probable anxiety or depression and somatic symptoms

Experiencing either of the main exposures was associated with probable anxiety or depression. Whereas experiencing BHA was also associated with moderate-to-severe somatic symptoms, experiencing discrimination was not. Furthermore, witnessing acts of discrimination was found to be associated with moderate-to-severe somatic symptoms, despite it not being associated with probable anxiety or depression. The influence witnessing acts of discrimination has on health and psychological distress may be complex and requires further research (particularly qualitative) to understand it in more depth.

### Job satisfaction and sickness absence

Regarding work-related outcomes, both witnessing and experiencing the main exposures were associated with low job satisfaction and long periods (minimum 2 weeks) of sickness absence – these associations persisted after accounting for long-standing health problems, illness or disability. Further research is needed to unpick the causal mechanisms underlying these associations and in particular to confirm whether discrimination and BHA lead to sickness absence either as a result of poor health or as a strategy adopted by staff to minimise exposure to harmful work environments.^43–46^ This is particularly pressing given that NHS staff sickness rates rose to 4.1% in April 2019, representing more than 1.4 million full-time days lost in that month alone.^47^

These findings echo those from previous studies that have examined the effects of discrimination and BHA in other types of workplaces, and have illustrated that both exposures are equally associated with negative health and work-related outcomes.^3,10^ This study also provides some evidence that witnessing such events, although not as detrimental as experiencing them, can have a negative impact on health, indicating that the impact of a hostile work environment is not limited to those being directly affected.

### Strengths and limitations

This study assesses NHS staff discrimination and BHA in London (the most prevalent region for these incidents), and how exposure to either has an impact on the health, well-being and job performance of staff. This is the first survey to investigate the experiences and well-being of NHS staff that is independent of the NHS. The sample has also been weighted to reflect the ethnic breakdown of HCPs working in London trusts (see Supplementary Table 1). The cross-sectional nature of the study design precludes any causal inferences regarding the associations found in this study. However, the TIDES survey data is part of a larger study and qualitative data from survey participants is being gathered to ensure that the experiences, training and reporting procedures staff undergo are understood in more depth.

The survey was administered online, and therefore susceptible to mode bias, although, as many participants require online access for their training and work, this is unlikely. Participation was voluntary, and as this survey was both confidential and independent of the NHS, HCPs who were particularly interested in the topic may have been more inclined to take part. The measure of long-standing health problems, illness or disability used in this study may have been an insufficient indicator of recent health. We are therefore unable to exclude the possibility that sickness absence was taken for an unaccounted-for health problem – further research is needed to determine whether this is the case or whether harassment and discrimination leads to voluntary sickness absence.

We acknowledge that the TIDES sample is younger than its target demographic as shown in Supplementary Table 1. This may limit the generalisability of our findings, but also provides evidence to inform interventions for HCPs at early stages in this occupational pipeline. The majority of people who completed the TIDES survey were nurses (64%), therefore results may reflect this occupation/environment rather than NHS staff overall. This study used measures of probable anxiety, depression and somatic symptoms that have been widely validated in a variety of populations and countries, and in similar samples to that of this study. It also used measures of discrimination and BHA that are comparable with WRES and national NHS survey data.

Finally, a key strength of this survey is that it is independent of the NHS – currently the only existing data on staff discrimination and harassment in the NHS is taken from the NHS staff survey (subsequently used by WRES), which is subject to biased responses from NHS employees who may be hesitant to declare their experiences in an employer administered survey. Although some TIDES survey gatekeepers were NHS staff, the survey itself was administers via the TIDES team and all derived data held and analysed by TIDES.

### Implications

Through the WRES, workplace inequality in the NHS is now closely monitored and scrutinised. However, since its inception, rates of discrimination and harassment have continued to rise, indicating that more is needed to address this issue.

NHS staff well-being should be paramount, yet HCPs (and by proxy, their patients) are being let down by individuals in leadership positions supporting a system that allows harassment and discrimination to occur without ramification. Priority should be given to providing support to members of staff most affected and using their experiences to shape NHS workforce policy going forward. This also means closely assessing the barriers for racial and ethnic minoritystaff obtaining more senior roles.

Safe spaces should be created to allow staff to discuss what they have experienced and witnessed in the workplace, particularly with regards to race, ethnicity and migrant status. Upscaling small-scale initiatives such as NHS's ‘freedom to speak up to guardians’ and various mentoring schemes could help – although these should be evaluated to determine the extent to which they support staff.

Equality, diversity and inclusion must be aligned throughout all levels of the NHS and in conjunction with the higher education sector where HCPs are clinically trained. Equality, diversity and inclusion training is important, but currently exists as infrequent dedicated training sessions (often e-learning modules) that is insufficient on its own. Multilevel, multisector strategies to enforce mandated diversity policy and interventions should be introduced and integrated more widely.^19^

To conclude, NHS staff, particularly those working in London trusts, are exposed to unprecedented levels of discrimination and BHA from their colleagues. It is only by understanding the impact this has on their health and job satisfaction that we can begin to take steps to mitigate this epidemic. This study – the first in the UK to examine associations between these exposures and both health and job satisfaction in NHS staff – has found discrimination as well as BHA committed by and against staff in the NHS to be both prevalent and harmful. Structural changes to the way staff are supported, and how their complaints can be addressed by leaders within the institution, are potential points of intervention to alleviate this issue. Within the context of an already stretched and under-resourced NHS, to combat poor job satisfaction and high turnover rates that ultimately have an impact on quality healthcare, the value of all HCPs must be visibly and continuously reinforced by all management and senior leaders.

## Data Availability

TIDES survey data is available upon request. Please email tides@kcl.ac.uk to apply for the data.

## Supplementary material

For supplementary material accompanying this paper visit http://dx.doi.org/10.1192/bjo.2020.137. click here to view supplementary material
