# Supplementary material for: Impact of workplace discrimination and harassment among National Health Service staff working in London trusts: results from the TIDES study
Source: BJPsych Open. 2020 Dec 16;7(1):e10. doi: 10.1192/bjo.2020.137 (PMC7791556; doi:10.1192/bjo.2020.137)
Supplement: Supplementary file 1 [file bjosup.zip › S2056472420001374sup001.docx]

**Supplementary Table 1: Weighted and unweighted TIDES survey sample – weights were generated using HR data from NHS London trusts**

|  |  | **TIDES Sample** | | | **NHS London Data** | |
| --- | --- | --- | --- | --- | --- | --- |
|  |  | **Unweighted** | | **Weighted for gender and ethnicity** |  |  |
|  |  | **n** | **%** | **%** | **n** | **%** |
| **Gender** | |  |  |  |  |  |
|  | Male | 167 | 17.9 | 24.4 | 42515 | 24.4 |
|  | Female | 764 | 82.1 | 75.6 | 131804 | 75.6 |
| **Age group (years)** | |  |  |  |  |  |
|  | <25 | 251 | 27.0 | 26.5 | 8188 | 4.8 |
|  | 25-34 | 449 | 48.2 | 47.8 | 52158 | 30.6 |
|  | 35-44 | 142 | 15.3 | 16.1 | 42982 | 25.2 |
|  | 45+ | 89 | 9.6 | 9.5 | 67303 | 39.4 |
| **Ethnicity** | |  |  |  |  |  |
|  | White British | 421 | 45.3 | 43.5 | 84464 | 54.46 |
|  | White Other | 107 | 11.5 | 10.9 |  |  |
|  | Black | 179 | 19.2 | 21.6 | 33531 | 21.62 |
|  | Asian | 166 | 17.8 | 20.5 | 31752 | 20.47 |
|  | Mixed | 57 | 6.1 | 3.5 | 5353 | 3.45 |
| **Migrant status** | |  |  |  |  |  |
|  | Non-migrant | 603 | 64.8 | 62.8 | - | - |
|  | Migrant | 328 | 35.2 | 37.2 | - | - |
| **Sexual Orientation** | |  |  |  |  |  |
|  | Heterosexual | 806 | 89.1 | 88.4 | - | - |
|  | Non-heterosexual | 99 | 10.9 | 11.6 | - | - |
| **Occupational group** | |  |  |  |  |  |
|  | Medical | 138 | 14.8 | 16.8 | - | - |
|  | Allied health professionals /Psych | 93 | 10.0 | 10.2 | - | - |
|  | Nurse | 600 | 64.4 | 64.5 | - | - |
|  | HCA | 75 | 8.1 | 8.30 | - | - |
|  | Other | 25 | 2.7 | 0.20 | - | - |
