# Supplementary material for: Impact of workplace discrimination and harassment among National Health Service staff working in London trusts: results from the TIDES study
Source: BJPsych Open. 2020 Dec 16;7(1):e10. doi: 10.1192/bjo.2020.137 (PMC7791556; doi:10.1192/bjo.2020.137)
Supplement: Supplementary file 1 [file bjosup.zip › S2056472420001374sup002.docx]

**Supplemental Document**

**TIDES survey recruitment procedure**

First, gatekeepers for accessing foundation and trainee doctors, nurses, healthcare assistants and IAPT workers were contacted to gain permission for the research study to be conducted. Gatekeepers included:

1. Foundation managers and training programme directors.
2. Department heads, preceptorship module leaders and programme directors.
3. IAPT Service managers.

Once initial gatekeeper consent was gained, participants were recruited via three pathways: (i) attending training or education sessions for healthcare practitioners/trainees, (ii) email circulars sent via relevant HEI programme directors and directors/managers in nursing, IAPT and foundation doctor training programmes and (iii) through social media and the study website. Individual HCPs interested in participating in the study contacted the researchers via email. Eligibility was assessed via email correspondence. Eligible participants were sent an information sheet and service guide, and if they agreed to take part, a link to the online survey was sent to them from Qualtrics. Consent forms were completed at the beginning of the online survey. Participants who completed the survey were compensated with a £15 e-voucher.

**How did gatekeepers select participants?**

Rather than selecting participants, gatekeepers supported us by advertising the TIDES study to eligible participants in the following ways:

- - For universities, lecturers and course leaders were asked to disseminate information on the TIDES study to undergraduate and/or postgraduate students (e.g. through course mailouts). Gatekeepers at all nursing and healthcare assistant programmes based in London universities were contacted.
  - For trusts, gatekeepers permitted the TIDES researchers to speak at existing team meetings to advertise the study directly.
  - In addition, administrators for foundation medical training programmes in all London trusts were asked to circulate TIDES flyers and study information.

**Were there any refusals to take part?**

Participation was entirely voluntary, and healthcare practitioners could only take part if they emailed TIDES researchers directly and asked for a unique link to the online survey. Gatekeepers had no way of knowing who did and did not take part in the survey. Two gatekeepers were unable to allow TIDES researchers to speak at their team meeting due to time constraints but instead circulated the TIDES study flyer via email. There were no refusals from gatekeepers to aid the study in any way.

**How was the survey introduced to potential participants?**

Where possible, research assistants from the TIDES study were invited to lectures/team meetings to give a short presentation about the study. Afterwards, further information about the survey and how to take part was circulated via email. Potential participants were asked to contact the TIDES team directly to receive a unique link to the online survey (which could not be shared between participants). This way, no one had to indicate their willingness to participate in front of their peers. It was made very clear that participation was entirely voluntary and refusal to take part would have no consequences. Due to the nature of the survey, we reassured potential participants that the study was independent from the NHS.

**Representativeness**

The aim of our study was to recruit a representative sample while also remaining impartial. This was particularly important as similar workforce surveys that are affiliated with the NHS have low response rates - largely due to concerns over confidentiality. The NHS staff survey for example (which is strongly advertised internally) typically has responses rates of around 40%, and studies such as NHS CHECK, who do engage with NHS HR, struggle with very low response rate (less than 20%) and reported concerns over trust and confidentiality from NHS staff. Our study reached out to all 34 London trusts and got participants from 33 – our researchers also worked hard to build relationships with these trusts and their staff, assuring participants that their responses were not only valued but would remain confidential. We also weighted our sample by gender and ethnicity (using HR data from participating trusts) to better reflect the study population and incorporate survey weights to all the analysis being conducted.

There was a bias towards recruiting early career HCPs from KHP trusts as we aim to inform local equality, diversity and inclusion initiatives (e.g., career progression) policies and KCL is one of only four nursing/HCA programmes in London (all of which engaged with the TIDES study during recruitment).
